# Prognostic risk factors of pneumonia associated with COVID-19 in patients with lymphoma

**DOI:** 10.3389/fonc.2024.1504809

**Published:** 2025-01-06

**Authors:** Dan Liu, Xia Yin, Hui Wang, Lijie Xing, Ping Li, Haichen Wei, Ji Ma, Qiang He, Linna Xie, Ke Lu, Zengjun Li

**Affiliations:** Department of Lymphoma, Shandong Cancer Hospital and Institute, Shandong First Medical University and Shandong Academy of Medical Sciences, Jinan, China

**Keywords:** COVID-19, pneumonia, lymphoma, bruton’s tyrosine kinase inhibitor, hematologic malignancy

## Abstract

**Objective:**

Patients with hematological malignancies have an elevated risk of developing pneumonia after contracting COVID-19. Lymphoma is the most prevalent hematologic malignancy. It is critical to identify patients at high risk of contracting COVID-19-associated pneumonia.

**Methods:**

From January 11–31, 2023, we distributed questionnaires to patients diagnosed with lymphoma according to 2016 World Health Organization diagnostic and classification criteria. COVID-19 infection was confirmed based on symptoms and laboratory tests. Pneumonia was confirmed using computed tomography scans.

**Results:**

In total, 257 patients were included in this study; 221 patients (86.0%) had COVID-19 infection and 61 (27.6%) of these had pneumonia. Patients with B-cell non-Hodgkin lymphoma (B-NHL) had a significantly higher pneumonia incidence than patients with other lymphoma types (31.8% vs. 27.6%, P=0.005). Higher incidence of pneumonia was observed in patients receiving anti-CD20 therapy (30.0% vs. 16.3%, P=0.048) and Bruton’s tyrosine kinase (BTK) inhibitor therapy (51.3% vs. 22.5%, P=0.001). B-NHL (hazard ratio [HR]=3.7, 95% confidence interval [CI] 1.4–10.0, P=0.009), anti-CD20 therapy (HR=2.3, 95% CI 1.0–5.2, P=0.050), BTK inhibitor (HR=3.6, 95% CI 1.8–7.4, P<0.001), active therapy (HR=3.0, 95% CI 1.5–5.7, P=0.001), and lack of disease remission (HR=3.7, 95% CI 1.8–7.4, P=0.001) were high-risk factors for developing pneumonia. Anti-PD-1 therapy was a protective factor against pneumonia development (HR=0.2, 95% CI 0.05–0.9, P=0.034). In multivariable analysis, BTK inhibitor (HR=3.5, 95% CI 1.6–8.0, P=0.003), active therapy (HR=3.3, 95% CI 1.6–6.8, P=0.001), and disease non-remission (HR=2.9, 1.3–6.4, P=0.007) were independent risk factors for pneumonia development after COVID-19 infection in patients with lymphoma.

**Conclusions:**

Patients with lymphoma receiving BTK inhibitors, undergoing active therapy, and lacking disease remission exhibited a higher risk for pneumonia associated with COVID-19.

## 1 Introduction

The COVID-19 pandemic that began in December 2019 was declared a global pandemic by the World Health Organization in March 2020. Patients with malignancies, particularly those with hematological malignancies, are highly susceptible to pneumonia and mortality following COVID-19 infection (1, 2) owing to anticancer therapy or malignancy itself. The mortality rate related to COVID-19 among patients with hematological malignancy is very high, reportedly 30% to 40% (3–5). However, these results are mostly based on hospitalized patients and were from the early period of the COVID-19 pandemic when the virus was more aggressive and medical resources were scarcer. Since that time, a considerable number of patients with cancer have experienced COVID-19 infection out of hospital and the mortality rate has decreased owing to weakened virulence of the causative virus, SARS-CoV-2, as well as better accessibility to anti-COVID-19 drugs. However, related reports are rare regarding outcomes among patients with cancer who develop COVID-19 infection.

Lymphoma is the most prevalent hematologic malignancy. Previous studies have primarily focused on identifying the mortality risk factors among patients with lymphoma following COVID-19 infection (1, 6). Reportedly, older age, cancer type, therapy, and complications are associated with mortality owing to COVID-19 infection among patients with lymphoma (4, 7). There is scant research examining the pneumonia incidence and associated risk factors within this patient cohort. China removed most COVID-19 quarantine measures in December 2022. Most individuals in China experienced their first COVID-19 infection between December 2022 and February 2023. We conducted a questionnaire survey targeting patients with lymphoma to investigate their experiences with COVID-19 infection and pneumonia occurrence during the epidemic wave involving the SARS-CoV-2 Omicron variant. This effort will help hematologists to recognize high-risk patients and improve preventive measurements.

## 2 Methods

### 2.1 Study design and participants

This was a single-center, retrospective study among patients with lymphoma who were treated or followed up at the Lymphoma Department of Shandong Cancer Hospital. A questionnaire was distributed to patients with lymphoma via WeChat from January 11, 2023; follow-up with these patients was carried out until September 30, 2023. The questionnaire included demographic information, complications, and questions regarding COVID-19 infection among patients, such as the date of infection, symptoms, occurrence of pneumonia, and therapy. Patients may have certain limitations in their memory regarding their COVID-19 infection history, which may lead to recall bias. Other information such as diagnosis, anti-tumor treatment, and disease status, were collected from the inpatient or outpatient medical records. All patients included in this study were diagnosed with lymphoma according to the World Health Organization 2016 diagnostic and classification criteria. Regarding treatment patterns, patients were treated according to NCCN guidelines, which aligns with the treatment regimens commonly used for lymphoma populations. All information was collected according to local data protection laws. We have implemented strict protocols to protect patient confidentiality, including de-identification of patient data before analysis. All identifying information has been removed from the dataset to ensure that individual responses cannot be traced back to the participants. Additionally, all data was stored securely in password-protected files, accessible only to authorized personnel. To address potential bias in patient-reported data, we employed several strategies: We used standardized assessment tools and questionnaires to minimize variability in responses. We provided clear instructions to participants to encourage accurate reporting and reduce misunderstanding. Regular training sessions were conducted for staff involved in data collection to reinforce the importance of objective patient engagement and data accuracy. We obtained study approval from the appropriate ethics committee.

COVID-19 infection was confirmed based on symptoms and laboratory tests, including reverse transcription polymerase chain reaction or antigen testing. In our study, patients were predominantly infected with Omicron strains. Pneumonia was confirmed using computed tomography scans conducted within 30 days from the onset of COVID-19 infection. Active therapy was defined as antitumor therapy delivered during hospital admission for COVID-19 infection or within the previous 3 months. Responses to lymphoma treatment were evaluated according to the Lugano criteria (2014) (8). We included laboratory parameters collected during COVID-19 infection or within 1 month before infection.

### 2.2 Statistical analysis

Quantitative variables are expressed as the median and (IQR). For descriptive analysis, Pearson’s chi-square and Mann–Whitney *U* tests were used to compare categorical and quantitative variables, respectively. Cox regression was used to infer pneumonia risk factors. For variables significantly related to pneumonia, multivariate analysis was performed. Statistical significance was defined as *P*<0.05. In the analysis, we used IBM SPSS version 26 (IBM Corp., Armonk, NY, USA).

## 3 Results

### 3.1 Patient characteristics

A total of 340 patients completed the questionnaire survey. In total, 29 patients were diagnosed with multiple myeloma and 15 with other hematologic diseases; 22 patients lacked detailed records, and 17 were lost to follow-up. Consequently, the final analysis included a cohort of 257 patients.

Among the 257 patients included in this study, 121 were female and 136 were male patients. The median patient age was 53 years (IQR 43–64). Twenty-two (8.6%) patients were diagnosed with Hodgkin’s lymphoma (HL) and 235 (91.4%) had non- Hodgkin’s lymphoma (NHL). Among patients with NHL,122 had diffuse large B-cell lymphoma, 34 had follicular lymphoma (FL), 14 had marginal zone lymphoma, eight had mantle cell lymphoma, eight had chronic lymphocytic leukemia, two had hairy cell leukemia, 26 had peripheral T-cell lymphoma, 13 had natural killer/T-cell lymphoma, and eight patients were diagnosed with other types of lymphoma. A total of 143 patients (55.6%) were undergoing treatment, and 114 (44.4%) patients were not receiving active treatment.

### 3.2 COVID-19 infection

**Figure 1:**
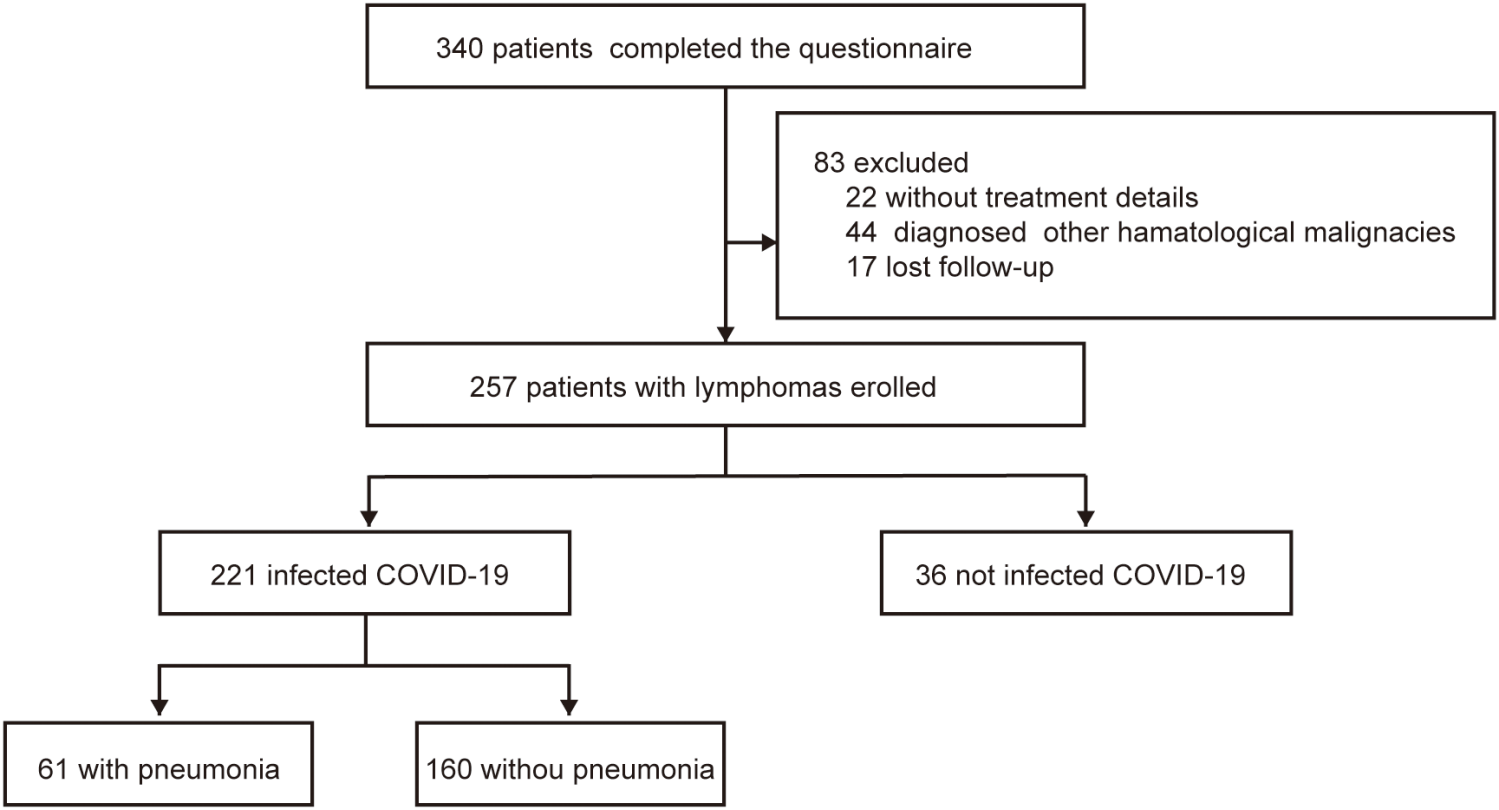
Study profile.

Up to the last follow-up in September 2023, 221 patients had been infected with COVID-19, and 36 patients had not been infected. COVID-19 infection was observed in 221 patients (86.0%); the remaining 36 patients (14.0%) were uninfected between November 11, 2022 and February 15, 2023 (**Figure 1**). The group of COVID-19 infection comprised 111 male (50.1%) and 110 female (49.1%; **Table 1**) patients. And the number of infection patients diagnosed with B-cell lymphoma, T-cell lymphoma, and HL was 176 (79.6%), 32 (14.5%), and 13 (5.9%), respectively. Fatigue (93.7%) and fever (86.9%) were the most common symptoms after COVID-19 infection (**Supplementary Figure 1**). Five (2.3%) patients died after infection with COVID-19. The mortality was much lower compared with previous studies among patients with lymphoma (100-day mortality 23%–35% after COVID-19 infection) (6, 9).

**Table 1:** Clinical and laboratory features of patients with and without Covid-19 infection.

	Infected (n=221)	Uninfected (n=36)	P-value
Age (years); median (IQR)	53 (43-64)	58 (48-67)	0.283
Female, n (%)	110 (49.8)	11 (30.6)	**0.047**
WBC, × 10E+9/L; median (IQR)	4.5 (3.2-5.9)	4.7 (3.5-5.7)	0.920
HB, × 10E+9/L; median (IQR)	126 (112-139)	135 (118-143)	0.186
PLT, × 10E+9/L; median (IQR)	196 (147-251)	223 (161-257)	0.452
Lym, × 10E+9/L; median (IQR)	1.0 (0.7-1.5)	1.1 (0.7-1.4)	0.823
ANC, × 10E+9/L; median (IQR)	2.6 (1.8-3.9)	2.64 (1.6-3.8)	0.874
Lymphoma types			0.001
B-NHL, n (%)	176 (79.6)	20 (55.6)	
T-NHL, n (%)	32 (14.5)	8 (22.2)	
HL, n (%)	13 (5.9)	8 (22.2)	
Anti-CD20 therapies, n (%)	172 (77.8)	20 (55.6)	**0.007**
Bendamustine, n (%)	32 (14.5)	3 (8.3)	0.436
Anti-PD-1 therapies, n (%)	25 (11.3)	7 (19.4)	0.177
BTK inhibitors, n (%)	39 (17.6)	6 (16.7)	1.000
Treatment lines			0.363
Untreated or first line, n (%)	169 (76.5)	25 (69.4)	
≥2 lines, n (%)	52 (23.5)	11 (30.6)	
Treatment stature, n (%)			0.721
Active therapies, n (%)	124 (56.1)	19 (52.8)	
No active therapy, n (%)	97 (43.9)	17 (47.2)	
Vaccinated, n (%)	145 (65.5)	28 (77.8)	0.181
Diabetes, n (%)	17 (7.7)	3 (8.3)	1.000
Hypertension, n (%)	38 (17.2)	6 (16.7)	1.000
Coronary heart disease, n (%)	10 (4.5)	3 (8.3)	0.402

WBC, white blood cell; HB, hemoglobin; PLT, platelet; Lym, lymphocyte count; ANC, absolute neutrophil count; NHL, non-Hodgkin lymphoma; HL: Hodgkin lymphoma; BTK, Bruton’s Tyrosine Kinase.

We compared the clinical characteristics of patients with and without COVID-19 infection and found that female patients had a greater likelihood of infection than their male counterparts (69.4% vs. 50.2%, P=0.047). Patients with NHL had a much higher infection rate than those with HL (88.1% vs. 61.9%, P=0.004). The group with COVID-19 infection included a higher proportion with B-NHL than patients with other types of lymphoma (79.6% vs. 55.6%, P=0.001; **Table 1**) and were more likely to have received anti-CD20 therapy (77.8% vs. 55.6%, P=0.007). There were no significant differences in COVID-19 infection rates among patients across treatment lines or between those receiving active treatment and those who were not. Univariate analysis showed that female sex (hazard ratio [HR]=2.25, 95% confidence interval [CI] 1.06–4.80, P=0.035), receiving anti-CD20 therapy (HR=2.81, 95% CI 1.35–5.83, P=0.006), and B-NHL (HR =3.13, 95% CI 1.50–6.52, P=0.002) were risk factors for COVID-19 infection (**Supplementary Table 1**). However, in multivariable analysis, we did not identify any independent risk factors among these three factors.

### 3.3 Pneumonia occurrence

Among the 221 patients infected with COVID-19, a total of 61 (23.7%) developed pneumonia associated with COVID-19. Comparing the clinical characteristics of patients with and without pneumonia, patients who developed pneumonia had a higher median age (58 vs. 52 years, P=0.034; **Table 2**), higher prevalence of B-cell lymphoma (91.8% vs. 75.0%, P=0.020), and were more likely to have previously received anti-CD20 therapy (86.9% vs. 74.4%, P=0.048) as well as Bruton’s tyrosine kinase (BTK) inhibitor therapy (32.8% vs. 11.9%, P=0.001). Additionally, patients with pneumonia demonstrated a greater frequency of receiving active therapy (75.4% vs. 48.8%, P<0.001) and of disease non-remission (34.4% vs. 12.5%, P<0.001). The proportion of patients receiving anti-PD-1 therapy was lower in those who developed pneumonia compared with patients who did not develop pneumonia (3.3% vs. 14.4%, P=0.018).

**Table 2:** Clinical and laboratory features of patients with or without pneumonia after Covid-19 infection.

	Pneumonia (n=61)	No pneumonia (n=160)	P-value
Age (years); median (IQR)	58 (46-67)	52 (40-61)	**0.034**
Male, n (%)	29 (47.5)	82 (51.2)	0.654
WBC, × 10E+9/L; median (IQR)	5.0 (3.1-6.7)	4.2 (3.2-5.7)	0.196
HB, × 10E+9/L; median (IQR)	123 (105-136)	128 (115-140)	0.095
PLT, × 10E+9/L; median (IQR)	198 (137-273)	195 (148-248)	0.946
Lym, × 10E+9/L; median (IQR)	1.0 (0.5-1.5)	1.1 (0.7-1.5)	0.280
ANC, × 10E+9/L; median (IQR)	2.9 (1.6-4.3)	2.6 (1.8-3.7)	0.285
Lymphoma types			0.020
B-NHL, n (%)	56 (91.8)	120 (75.0)	
T-NHL, n (%)	3 (4.9)	29 (18.1)	
HL, n (%)	2 (3.3)	11 (6.9)	
Anti-CD20 therapies, n (%)	53 (86.9)	119 (74.4)	0.048
Bendamustine, n (%)	13 (21.3)	19 (11.9)	0.088
Anti-PD-1 therapies, n (%)	2 (3.3)	23 (14.4)	**0.018**
BTK inhibitors, n (%)	20 (32.8)	19 (11.9)	**0.001**
Treatment lines			0.596
Untreated or first line, n (%)	45 (73.8)	124 (77.5)	
≥2 lines, n (%)	16 (26.2)	36 (22.5)	
Treatment stature, n (%)			<0.001
Active therapies, n (%)	46 (75.4)	78 (48.8)	
No active therapy, n (%)	15 (24.6)	82 (51.2)	
Response			<0.001
Lack remission, n (%)	21 (34.4)	20 (12.5)	
Remission, n (%)	40 (65.5)	140 (87.5)	
Vaccinated, n (%)	47 (77.0)	98 (61.3)	**0.028**
Diabetes, n (%)	4 (6.6)	13 (8.1)	0.786
Hypertension, n (%)	13 (21.3)	25 (15.6)	0.324
Coronary heart disease, n (%)	3 (4.9)	7 (4.4)	1.000

WB, white blood cell; HB, hemoglobin; PLT, platelet; Lym, lymphocyte count; ANC, absolute neutrophil count; NHL, non-Hodgkin lymphoma; HL, Hodgkin lymphoma; BTK, Bruton’s Tyrosine Kinase.

**Figure 2:**
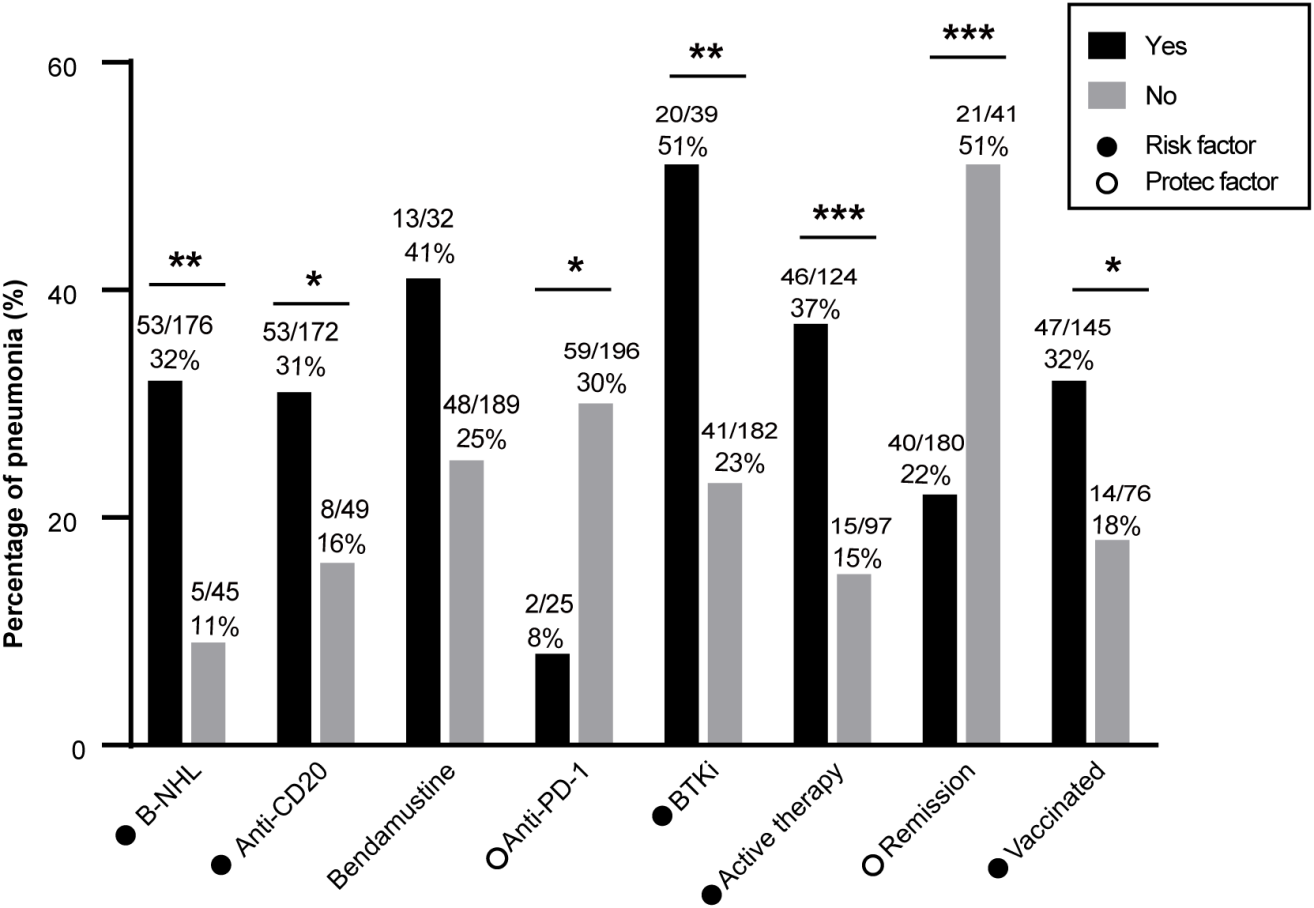
Pneumonia incidence in patients with lymphoma after COVID-19 infection. B-NHL, anti-CD20 therapy, BTKi, active treatment, lack of disease remission, and vaccination were associated with higher pneumonia incidence. Anti-PD-1 therapy was associated with a lower rate of pneumonia. B-NHL, B-cell non-Hodgkin lymphoma; HL, Hodgkin lymphoma, BTKi, Bruton’s tyrosine kinase inhibitor. *: P<0.05; **: P<0.01; ***: P<0.001.

The incidence of COVID-19 pneumonia was analyzed in subgroups. Patients with B-cell lymphoma exhibited a significantly higher incidence of pneumonia compared with patients who had other types of lymphoma (31.8% vs. 27.6%, P=0.005; **Figure 2**). A higher incidence of pneumonia was observed in patients who previously received anti-CD20 therapy (30.0% vs. 16.3%, P=0.048) and BTK inhibitor therapy (51.3% vs. 22.5%, P=0.001). Conversely, patients who received anti-PD-1 therapy demonstrated a lower incidence of pneumonia (8.0% vs. 30.1%, P=0.018).

Active treatment was associated with a significantly higher incidence of pneumonia compared with not undergoing treatment (37.1% vs. 15.5%, P<0.001). Furthermore, patients who did not achieve remission had a significantly higher incidence of pneumonia (51.2% vs. 22.2%, P<0.001). In total, 135 patients (65.6%) had received a COVID-19 vaccine. However, the vaccine protection was very limited, and vaccinated patients displayed a higher incidence of pneumonia compared with unvaccinated patients (32.4% vs. 18.4%, P=0.028).

Obinutuzumab and rituximab are both recommended as first-line treatment in FL according to National Comprehensive Cancer Network guidelines. A previous study found that patients who were receiving obinutuzumab had a higher risk of severe COVID-19 infection than those treated with rituximab (10). We compared the pneumonia incidence between receiving treatment with obinutuzumab and with rituximab in patients with FL. The pneumonia incidence was not significantly different (P=0.377) between patients with FL who were treated with obinutuzumab (33.3%, 4/12) and those treated with rituximab (14.3%, 3/21).

### 3.4 Risk factors of pneumonia associated with COVID-19

**Figure 3:**
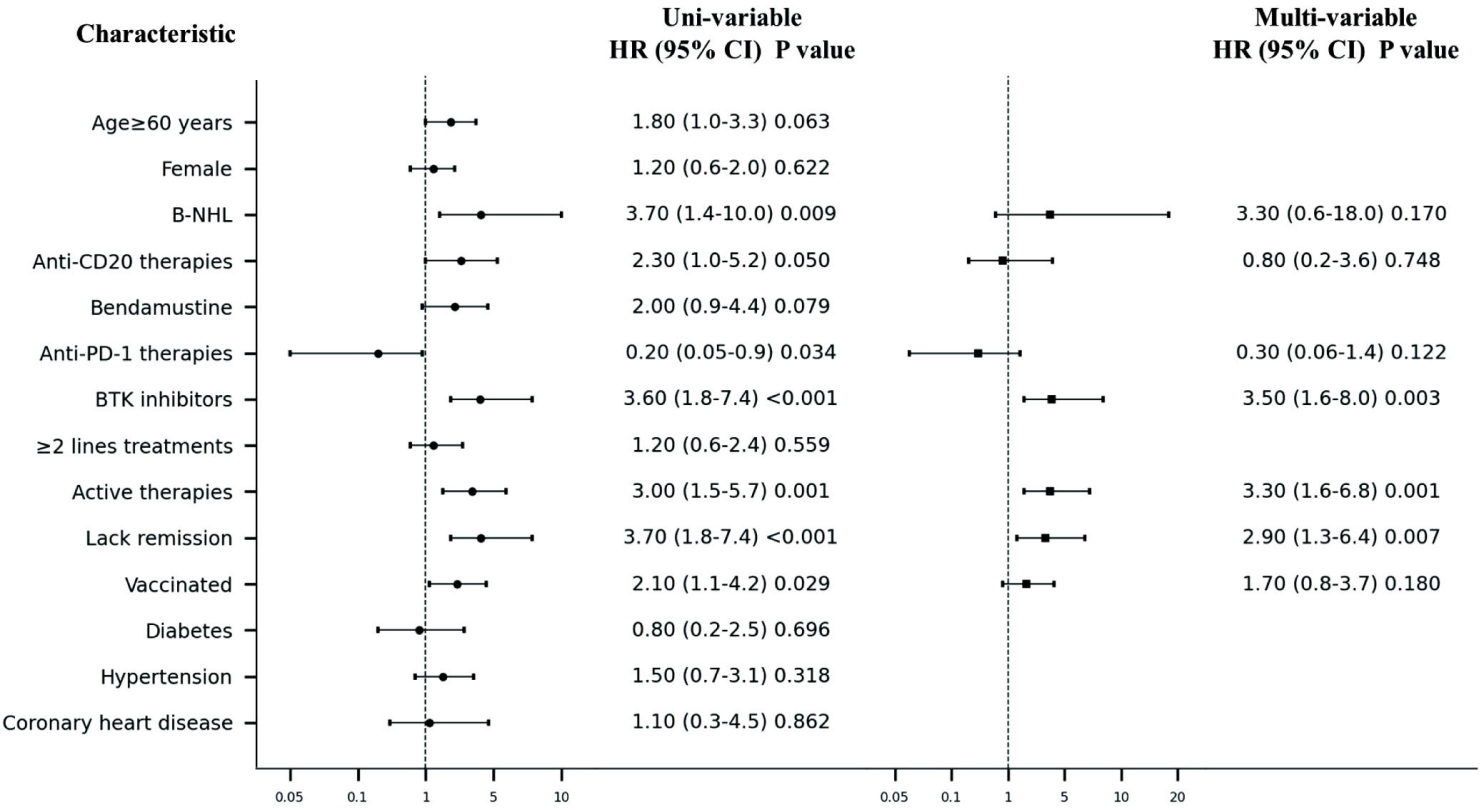
Forest plot of Uni- and multi-variable analysis of pneumonia after Covid-19 infection. NHL, non-Hodgkin lymphoma; BTK, Bruton’s Tyrosine Kinase.

In univariate analysis conducted using a logistic regression model, B-NHL (HR=3.7, 95% CI 1.4–10.0, P=0.009; **Figure 3**), anti-CD20 therapy (HR=2.3, 95% CI 1.0–5.2, P=0.050), BTK inhibitor (HR=3.6, 1.8–7.4, P<0.001), active therapy (HR=3.0, 1.5–5.7, P=0.001), and lack of disease remission (HR=3.7, 1.8–7.4, P=0.001) were high-risk factors for developing pneumonia in our study population. Conversely, anti-PD-1 therapy was found to be a protective factor against pneumonia development (HR=0.2, 95% CI 0.05–0.9, P=0.034). In contrast to findings in the general population where comorbidities such as hypertension and diabetes are known risk factors for adverse outcomes after COVID-19 infection (11), our study did not identify these conditions to be associated with an increased risk for developing pneumonia.

In the multivariable logistic regression model, BTK inhibitor (HR=3.5, 1.6–8.0, P=0.003), active therapy (HR=3.3, 95% CI 1.6–6.8, P=0.001), and disease non-remission (HR=2.9, 1.3–6.4, P=0.007) were identified as independent risk factors for the development of pneumonia after COVID-19 infection among patients with lymphoma.

## 4 Discussion

Before exploring the relationship between lymphoma patients and COVID-19 patients with viral pneumonia, the pneumonia-related adverse reactions during the treatment process should also be taken into account. Interstitial pneumonia is a lethal complication in lymphoma patients undergoing chemotherapy. A retrospective study found that the incidence of interstitial pneumonia was 3.9% (7/287) in patients with HL and 2.4% (76/1925) in patients with NHL (12). Furthermore, an analysis of the Chinese subgroup in the Phoenix study revealed that, compared to the R-CHOP group, combining the BTK inhibitor ibrutinib with R-CHOP increased the incidence of pneumonia-related adverse reactions from 2.1% to 9.7% (13). A study on the pulmonary adverse effects caused by the anti-CD20 monoclonal antibody rituximab reported that among all side effects, the incidence of pulmonary diseases may be the highest (14).

COVID-19 has been endemic since 2019, and the virulence of the virus has decreased substantially. However, patients with lymphoma remain at high risk for COVID-19 infection and subsequent pneumonia. Evidence from previous studies suggests that patients with cancer remain susceptible to comorbidity and mortality if infected with COVID-19, despite having received full vaccination (15, 16). Patients with lymphoma, especially those receiving anti-CD20 therapy, have poor or no vaccine response compared with healthy controls (17, 18). A meta-analysis reported a response rate of 64% for patients with hematologic malignancies following complete COVID-19 vaccination (19). Moreover, the response rates vary among specific types of lymphoma, with 91% for HL but only 58% for aggressive NHL and 61% for indolent NHL (19). Although most patients with NHL have been fully vaccinated, they remain susceptible to COVID-19 infection and developing pneumonia. The vaccination rate in our study was 67%; however, the protective effect against infection was found to be very limited. The vaccination-related findings seem contradictory. Vaccination can provoke an immune response; however, for patients with underlying diseases such as lymphoma, particularly those who have undergone chemotherapy or immunotherapy, the immune function may be significantly impaired, which can lead to dysregulation of the immune response and make them more susceptible to pneumonia. Indeed, we compared the differences in age, gender, lymphoma type, treatment received, and remission status between patients who received the vaccine and those who did not. We found that among vaccinated patients, a higher proportion were currently active therapies (59.5%) (**Supplementary Table 1**). Therefore, the increased pneumonia rate among vaccinated patients may be due to the fact that most patients were in the treatment process, leading to a decline in immune function. Additionally, vaccination may lead to increased healthcare engagement and monitoring among patients, which could mean that vaccinated patients are more frequently diagnosed with pneumonia, regardless of whether the vaccine truly affects the incidence.

Patients who received their last anti-CD20 treatment within 3 months of vaccination had a much lower response rate of 16% (18). These findings indicate that patients with HL exhibit a superior vaccine response compared with those who have NHL. This observation may potentially explain the lower incidence of pneumonia among patients receiving anti-PD-1 therapy; most individuals in this study who underwent such treatment were diagnosed with HL. Although our results suggest that anti-PD-1 therapy may have protective effects, the small sample size does pose a significant limitation to the generalizability of our findings. Further exploration in larger studies and meta-analyses is needed to validate our findings and better understand the true impact of anti-PD-1 therapy.

During the early stage of the COVID-19 pandemic, mortality was reported to be as high as 20%–40% among patients with cancer (6, 7, 9); therefore, the risk factors reported in previous studies for this population were nearly all for mortality. Older age, disease progression, indolent NHL, and aggressive NHL have been reported as risk factors for mortality in hematological malignancies (4, 7). Among hospitalized patients, cardiovascular disease, a lack of disease remission, active treatment, and CAR T-cell therapy are independently associated with mortality (20). Anti-CD20 treatments have also been found to independently influence prolonged viral detection among patients with hematologic malignancies (HR=3.64, 95% CI 1.44–8.97) (20). However, an Italian study reported that anti-CD20 therapy did not affect survival because this type of therapy does not impair cellular responses (9).

The mortality associated with COVID-19 was vastly decreased in our study compared with that in previous reports (6, 7, 9). This is partly because of increased access to antiviral drugs and decreased virulence of SARS-CoV-2. In a cohort of 308 pediatric patients with leukemia or lymphoma, 36% developed COVID-19 and only 6% patients developed severe disease (21). But the incidence of pneumonia was still high. Therefore, during the later stages of the COVID-19 pandemic, it is crucial to prioritize vigilance regarding pneumonia rather than just focusing on mortality. We found that anti-CD20 therapy and a lack of remission were related to a higher risk of pneumonia after infection with COVID-19, which was coincident with the mortality risk factors reported previously (22). A past study reported that the BTK inhibitor ibrutinib may provide protection against lung injury in patients with COVID-19 because it reduces proinflammatory and chemoattractant cytokines (23). However, that study lacked a large sample, and only included six patients with Waldenström macroglobulinemia who were receiving ibrutinib. In our study, BTK inhibitor was an independent risk factor for the occurrence of pneumonia after COVD-19 infection.

BTK inhibitors also increased common pneumonia by previous studies (13, 24, 25). In a multicenter, open-label phase II study assessing the efficacy and safety of ibrutinib in previously treated marginal zone lymphoma (MZL), grade 3-4 pneumonia was the most common adverse event (26). Additionally, an analysis of the Chinese subgroup in the Phoenix study found that the incidence of pneumonia-related adverse reactions increased from 2.1% to 9.7% when comparing the BTK inhibitor ibrutinib combined with R-CHOP to the R-CHOP group (13). In studies involving zanubrutinib for various B-cell malignancies, serious treatment-emergent adverse events included pneumonia at a rate of 11% (27). Furthermore, a phase II clinical study found that the incidence of grade ≥3 pneumonia in patients treated with acalbrutinib for mantle cell lymphoma was 5% (28). A pharmacovigilance study based on the FAERS database revealed that infection-related adverse reactions, such as pneumonia associated with ibrutinib and acalbrutinib, were among the most common safety signals linked to high mortality associated with these two BTK inhibitors (24).

Research shows that weighted gene co-expression network analysis combined with the explainable artificial intelligence algorithm LIME can comprehensively characterize the transcriptional changes in bronchial epithelial cells (including primary human bronchial epithelial cells and transformed alveolar cells) during severe acute respiratory syndrome coronavirus 2 (SARS-CoV-2) infection, and identify new hub gene features such as PGLYRP4 and HEPHL1 (29). Therefore, future studies can focus on the integration of COVID-19, SARS-CoV-2, bronchial epithelial cells, and transcriptomic analysis in lymphoma patients, especially regarding the gene networks associated with pneumonia risk when using BTK inhibitors or anti-CD20 therapies.

The present research was a retrospective study conducted at a single center. In terms of demographic characteristics, our study population primarily consisted of patients aged 43 to 64 years, with males accounting for 52.91% and females for 47.09%. Among the patients, 91.8% had NHL, while approximately 8.2% had HL. This is consistent with the overall incidence rates reported in a study on the burden of lymphomas in China in 2016. The study estimated that there were 6,900 new cases of HL and 68,500 cases of NHL in China. NHL has a higher incidence in the age group of 40 to 70 years, while the incidence of HL gradually increases after the age of 45, with males having a higher incidence than females (30). Further confirmation of our findings in large-scale multicenter cohort studies is warranted.

Our findings indicated that during the Omicron era of COVID-19, approximately 24% of our patients with lymphoma were found susceptible to developing pneumonia following viral infection, but with low mortality. Patients receiving BTK inhibitors, those undergoing active therapy, and those without disease remission exhibited a higher risk for developing pneumonia. So, for lymphoma patients, particularly those with B-cell non-Hodgkin lymphoma, especially those receiving BTK inhibitors or anti-CD20 therapy, it is recommended to implement personalized protective measures. This includes maintaining good hygiene practices during periods of high COVID-19 prevalence, providing education about the risks of COVID-19 and pneumonia, and encouraging patients to seek medical attention promptly when relevant symptoms arise.

## Data Availability

The original contributions presented in the study are included in the article/**Supplementary Material**. Further inquiries can be directed to the corresponding author.
