# Supplementary material for: Prognostic risk factors of pneumonia associated with COVID-19 in patients with lymphoma
Source: Front Oncol. 2025 Jan 6;14:1504809. doi: 10.3389/fonc.2024.1504809 (PMC11743689; doi:10.3389/fonc.2024.1504809)
Supplement: Supplementary file 1 [file Table1.docx]

**Supplementary**

**Supplementary Table 1.** **Uni- and multi-variable analysis of Covid-19 infection in patients with lymphoma.**

|  | Uni-variable | | | Multi-variable | | |
| --- | --- | --- | --- | --- | --- | --- |
|  | HR | 95%CI | P-value | HR | 95%CI | P-value |
| Age$\geq$60 years | 0.7 | 0.3-1.4 | 0.313 |  |  |  |
| Female | 2.3 | 1.1-4.8 | **0.035** | 2.1 | 1.0-4.6 | 0.051 |
| B-NHL | 3.1 | 1.5-6.5 | **0.002** | 2.7 | 0.4-16.6 | 0.288 |
| Anti-CD20 therapies | 2.8 | 1.4-5.8 | **0.006** | 1.1 | 0.2-7.0 | 0.893 |
| Bendamustine | 1.9 | 0.5-6.4 | 0.326 |  |  |  |
| Anti-PD-1 therapies | 0.5 | 0.2-1.3 | 0.176 |  |  |  |
| BTK inhibitors | 1.1 | 0.4-2.7 | 0.886 |  |  |  |
| Active therapies | 1.1 | 0.6-2.3 | 0.709 |  |  |  |
| Non-remission | 0.9 | 0.4-2.3 | 0.899 |  |  |  |
| Vaccinated | 0.5 | 0.2-1.2 | 0.154 |  |  |  |
| Diabetes | 0.9 | 0.3-3.3 | 0.894 |  |  |  |
| Hypertension | 1.0 | 0.4-2.7 | 0.938 |  |  |  |
| Coronary heart disease | 0.5 | 0.1-2.0 | 0.341 |  |  |  |

Abbreviations: NHL: non-Hodgkin lymphoma; HL：Hodgkin lymphoma；BTK: Bruton’s Tyrosine Kinase.

a: including treatment-naïve patients and those who have completed their treatment. b: including disease stable and progressed patients and treatment-naïve patients. c: including patients achieved part or complete remission.
