# Supplementary material for: Prognostic risk factors of pneumonia associated with COVID-19 in patients with lymphoma
Source: Front Oncol. 2025 Jan 6;14:1504809. doi: 10.3389/fonc.2024.1504809 (PMC11743689; doi:10.3389/fonc.2024.1504809)
Supplement: Supplementary file 2 [file Table2.docx]

**Supplement Table 2. Clinical and laboratory features of patients with or without vaccinated**

|  | Vaccinated, n (%) | unvaccinated, n (%) | P-value |  |
| --- | --- | --- | --- | --- |
| Age (years); median (IQR) | 53 (43-64) | 58 (48-67) | 0.283 |  |
| Sex |  |  | 0.904 |  |
| Female, n (%) | 81(46.8) | 40(47.6) |  |  |
| Male, n (%) | 92(53.2) | 44(52.4) |  |  |
| WBC, × 10E+9/L; median (IQR) | 4.3(3.1-5.9) | 4.6(3.5-5.6) | 0.825 |  |
| HB, × 10E+9/L; median (IQR) | 126.0 (111.2-138.7) | 132 (116.2-142.0) | 0.122 |  |
| PLT, × 10E+9/L; median (IQR) | 202.0(149.2-255.7) | 190.5(135.7-245.5) | 0.200 |  |
| Lym, × 10E+9/L; median (IQR) | 0.97(0.65-1.4) | 1.17 (0.83-1.5) | 0.089 |  |
| ANC, × 10E+9/L; median (IQR) | 2.5 (1.68-3.8) | 2.8 (1.9-3.8) | 0.749 |  |
| Lymphoma types |  |  | 0.521 |  |
| B-NHL, n (%) | 135 (78.0) | 61 (72.6) |  |  |
| T-NHL, n (%) | 26 (15.0) | 14(16.7) |  |  |
| HL, n (%) | 12 (6.9) | 9(10.7) |  |  |
| Anti-CD20 therapies, n (%) | 131 (75.7) | 61 (72.6) | 0.591 |  |
| Bendamustine, n (%) | 21 (12.1) | 14(16.7) | 0.321 |  |
| Anti-PD-1 therapies, n (%) | 13(7.5) | 19(22.6) | **0.001** |  |
| BTK inhibitors, n (%) | 27 (15.6) | 18 (21.4) | 0.249 |  |
| Treatment lines |  |  | **<0.001** |  |
| Untreated or first line, n (%) | 142(82.1) | 52(61.9) |  |  |
| $\geq$2 lines, n (%) | 31(17.9) | 32(38.1) |  |  |
| Treatment stature, n (%) |  |  | 0.071 |  |
| Active therapies, n (%) | 103(59.5) | 40(47.6) |  |  |
| No active therapy, n (%) | 70(40.5) | 44(52.4) |  |  |
| Diabetes, n (%) | 15(8.7) | 5(6.0) | 0.445 |  |
| Hypertension, n (%) | 36(20.8) | 8(9.5) | **0.024** |  |
| Coronary heart disease, n (%) | 9(5.2) | 4(4.8) | 0.880 |  |

Abbreviations: WBC: white blood cell; HB: hemoglobin; PLT: platelet; Lym: lymphocyte count; ANC: absolute neutrophil count; NHL: non-Hodgkin lymphoma; HL：Hodgkin lymphoma；BTK: Bruton’s Tyrosine Kinase.
