# Supplementary figures and images for: Prognostic risk factors of pneumonia associated with COVID-19 in patients with lymphoma
Source: Front Oncol. 2025 Jan 6;14:1504809. doi: 10.3389/fonc.2024.1504809 (PMC11743689; doi:10.3389/fonc.2024.1504809)

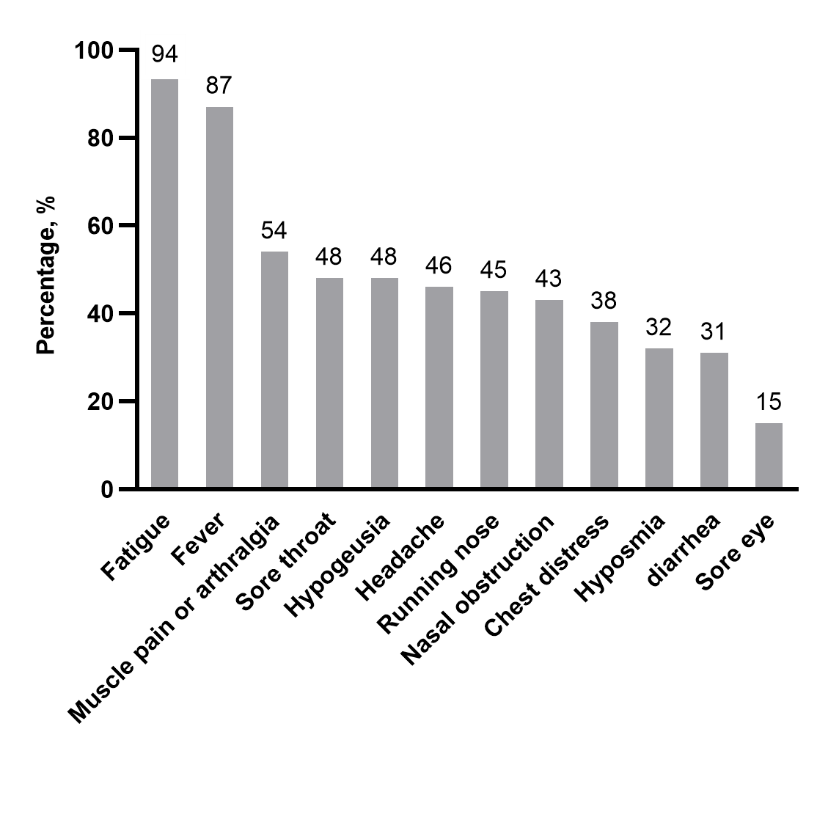


**Supplementary Figure 1. Symptoms in patients with lymphoma after Covid-19 infection**

Supplement: Supplementary file 3 [file DataSheet1.docx]
